# Case Report: Remission of schizophrenia using a carnivore ketogenic metabolic therapy with nutritional therapy practitioner support

**DOI:** 10.3389/fnut.2025.1591937

**Published:** 2025-06-24

**Authors:** Moira Newiss

**Affiliations:** Registered Nutritional Therapy Practitioner, Oban, United Kingdom

**Keywords:** schizophrenia, carnivore diet, ketogenic diet, ketogenic metabolic therapy, metabolic psychiatry, nutritional therapy, mood disorders, KMT

## Abstract

This retrospective case report presents the use of a carnivore ketogenic diet by a subject with schizophrenia, supported by a nutritional therapy practitioner, resulting in remission. The narrative describes how ketogenic metabolic therapy can be implemented and optimized in difficult socio-economic circumstances, something not previously reported in the literature. Compliance with diet is reported using glucose and ketone blood markers. The qualitative impact of the therapy is explored from the subject’s perspective as well as the potential for collaboration between nutritional and mental health practitioners to help implement and sustain ketogenic therapies.

## 1 Introduction

### 1.1 Schizophrenia prevalence and impact

Schizophrenia is a major psychiatric disorder affecting around 1% of the global population with a higher prevalence in males and peak onset usually in late teenage years or early adulthood^1^. Its diagnostic symptoms include those on the positive scale including delusions, grandiosity, and hallucinations, and those on the negative scale such as social withdrawal, anhedonia, poor rapport, blunted affect as well as cognitive symptoms which may involve problems with attention, memory, and decision-making (1, 2). The disorder exhibits a remitting and relapsing course, with varying degrees of recovery, and can significantly impact an individual’s ability to function leading to reduced quality of life, disability, and increased mortality rates (3, 4).

### 1.2 Schizophrenia and metabolic health

Attention has been recently directed toward the co-morbidity of schizophrenia and metabolic health conditions (4) and the underlying pathophysiological mechanisms at play leading researchers to propose that the condition be reconceptualized as impaired dynamic metabolic flexibility with bioenergetics failure (5). People with schizophrenia have a significantly higher incidence of obesity and prevalence of diabetes of 10–15%, which is a 2–3 times higher risk than in the general population (6, 7). Signs of metabolic syndrome were first identified in patients with schizophrenia over 100 years ago and current day use of antipsychotic medication comes with a major side effect of increasing metabolic dysregulation (8).

Underlying biological mechanisms identified in schizophrenia include oxidative stress, insulin resistance, glucose brain hypometabolism, mitochondrial dysfunction, inflammation, and glutamate/GABA imbalances (8, 9).

### 1.3 Ketogenic metabolic therapy

Ketogenic Metabolic Therapy (KMT) involves the prescription of a therapeutic ketogenic diet with a specific macronutrient distribution that is high in fat, moderate in protein and very low in carbohydrates (10). It enables the body to switch metabolism from running primarily on glucose to using fat as the primary fuel source (11). A ketogenic diet enables the body to enter a metabolic state known as “ketosis” when the liver starts to convert dietary fat into ketone bodies which are then circulated via the blood. At the cellular level ketones are taken into the mitochondria and used for oxidative phosphorylation to produce adenosine triphosphate (ATP) (12).

The definition of nutritional ketosis is ketones levels over 0.5 mmol/L (13), at these levels there is a growing body of evidence that the underlying biological processes relating to serious mental illness can be therapeutically impacted resulting in reduced symptoms and better mental wellbeing (9).

All the above mechanisms identified in schizophrenia can potentially be targeted with KMT which can reduce oxidative stress and inflammation, improve mitochondrial function, increase insulin sensitivity, enhance brain energy metabolism, increase GABA, and decrease glutamate (9, 14).

Relating to schizophrenia there are clinical trials and case reports describing the use of a ketogenic diet to improve symptoms and in some cases put schizophrenia into remission. Danan et al. (15) reported that 10 patients with a primary diagnosis of schizophrenia who followed a ketogenic diet for two weeks exhibited improvement in PANSS scores with clinically significant results. Two case reports from Palmer et al. (16) of 2 women who experienced complete remission of psychotic symptoms on a ketogenic diet for years after discontinuing anti-psychotic medication (16). A 4-month trial of 23 participants by Sethi et al. (17) found that 100% of individuals with a diagnosis of either schizophrenia or bipolar disorder who were compliant with the ketogenic diet achieved full recovery. Laurent et al. (18) present a case series of 2 individuals with schizophrenia, both of whom achieved complete cessation of psychotic symptoms and improvements in mood following a ketogenic diet which was validated using tools, including the Generalized Anxiety Disorder-7 (GAD-7), Depression Anxiety Stress Scales (DASS-42), and the PTSD Checklist for DSM-5 (PCL-5).

### 1.4 Schizophrenia and mitochondrial health

Neurons are dependent on mitochondria for energy (ATP) production and involved with Ca2 + homeostasis, apoptosis, brain development, and brain function (19). Studies show that the health of mitochondria, the efficiency of their ability to carry out oxidative phosphorylation, the ability to buffer Ca2 + and neutralize ROS, are all affected in schizophrenia (20). A study that investigated mitochondrial electron transport chain complex activity and protein and lipid oxidation markers in 11 subjects with schizophrenia found that mitochondrial complex I dysfunction and oxidative stress may play an important role in the pathophysiology of schizophrenia (21). Another study involving 82 schizophrenia patients measured markers of oxidative stress and found they may be associated with the severity of schizophrenia symptoms in positive, negative, and cognitive dimensions (22). Sarnyari and Ben-Shachar (5) reviewed the literature on central bioenergetics in schizophrenia focusing on altered glycolysis and mitochondrial oxidative phosphorylation finding abnormalities present from the first episode of psychosis and proposing approaches to influence energy metabolism and target mitochondrial health including use of KMT (5).

### 1.5 Carnivore ketogenic diets and mental health

A carnivore ketogenic diet is purely made up of meat and fat. This is the first case report of a carnivore ketogenic diet putting schizophrenia into remission. There is very limited research on carnivore diets in the medical literature especially in relation to carnivore or animal-based diets. One systematic review looking at associations between meat consumption and avoidance found that majority of higher quality studies showed individuals who avoided meat consumption had significantly higher rates of depression, anxiety, and/or self-harm behaviors (23). A later review examined literature on the relation between meat consumption or meat abstention and positive psychological functioning found mixed results with a small minority of studies showing meat consumers had a more positive psychological functioning, but no studies suggesting that meat abstainers did Dobersek et al. (24). An Australian study found that women consuming red meat within recommended levels had significantly lower risk of mood and anxiety disorders compared to those who ate lower amounts (25). A study of 2,029 people conducted using a social media survey of people self-identifying as following a strict carnivore diet found that 95% reported improvements in mental clarity, mood, and anxiety and most reported resolution of pre-existing psychiatric symptoms without medication (26).

## 2 Clinical background

The subject was a 32 year old male who was first diagnosed with schizophrenia 3 years previously when he developed psychotic behaviors. He first consulted the nutritional therapy practitioner after his recent discharge from a rehabilitation hospital. He was taking prescribed medication including amisulpride 600 mg, mitrazapine 40 mg and propanalol 80 mg. Over the past four years he had experienced mood instability along with paranoid delusions and suicidal thoughts. It had impacted severely on his daily life resulting in him being unable to work, finding it difficult to sustain relationships, and becoming homeless. On several occasions psychotic episodes resulted in him being sectioned and admitted to acute inpatient psychiatric care. The conventional treatment did not fully resolve his symptoms and his experience of the mental health service was not positive. He felt that no effort was made to address the underlying biological mechanisms driving his disease and he remained unable to functional normally in society.

The trigger for the onset of mental illness included the death of a close relative which was traumatic and unexpected. As a result, he had to move out of the family home to a new geographic location and started using cannabis as a coping strategy. He began experiencing negative thoughts, his behavior became more erratic, and he started experiencing psychotic episodes. This resulted in him becoming homeless and a family member raising the alarm to social services. Over the next 3 years he was in and out of hospital staying in homeless shelters in between these periods.

The subject was initially diagnosed with paranoid schizophrenia and subsequently additionally post-traumatic stress disorder. Whilst in hospital the main treatments he was given were medication, group psychology and occupational therapy, the latter of which consisted of trips to cafes, museums, and the library. He did not feel this had helped his mental health and that if anything that it deteriorated over the time that he was an in-patient.

The subject discovered the ketogenic diet when he read a self-help book during one of his early episodes living in a homeless shelter where it was suggested strategy to help improve your life. He decided to try a ketogenic diet simply to try and change his life, not with any expectation it would help his mental health. In the homeless shelter he had his own room where he was able to have a portable grill and minifridge and he adopted a carnivore ketogenic style of eating. However, at this stage he followed advice which allowed him to have ‘cheat days’ and on these days, he would eat large amounts of chocolate and other high sugar foods. He noticed that he felt better while eating foods that were ketogenic, but cheat days coincided with problems with his mood and behavior.

Over the period of the next few years, he continued research the ketogenic diet reading up about how it might help brain energy and how it might be a therapeutic metabolic treatment for various mental disorders. He decided to pursue ketogenic metabolic therapy as an alternative treatment as soon as he was able to. Whilst in acute hospital settings it was impossible to do so, however his last hospital placement was a rehabilitation facility where residents could cook for themselves. He was able to restart a ketogenic diet successfully eating foods like eggs, with some vegetables and fatty meat. Although his mental health support team were interested in what he was doing he received no support from them specifically about KMT.

On his discharge from hospital, he contacted a nutritional therapy practitioner who specialized in ketogenic metabolic therapy for mental illness with the aim of optimising his diet. He wanted help to get his psychiatrist on board with using KMT as a treatment to help him reduce his medication. At initial consultation the subject’s glucose and ketones levels were analyzed as well as his diet which was now 100% carnivore. His blood glucose and ketone readings were fluctuating greatly so the aim was to stabilize his glucose levels and optimize his ketones in the therapeutic range. In addition, the practitioner provided information about KMT to his consultant psychiatrist including research supporting the use of a ketogenic diet for serious mental illness. Throughout this period the subject had regular weekly contact with his local community mental health team.

At the first visit the subject reported feeling worried, a fight or flight feeling that would not go away, he was finding it difficult to make decisions and feeling anxious about his health. He reported dizziness and had on one occasion collapsed requiring a trip to the accident and emergency department. He reported weight loss and was concerned about losing any more weight. His current weight was 69 kg and BMI of 21.5. His eyes were sensitive to light, skin was slow to heal, and he was bothered by excess saliva (a side effect of his medication). His glucose and ketone levels were variable, glucose between 1 mmol/L and 6 mmol/L and ketones between 0 mmol/L and 12 mmol/L. His prior blood readings showed that he was not in a consistent or safe level of ketosis which was due to periods of prolonged fasting (27).

He reported that previous episodes of binge eating carbohydrates on “cheat days” had adversely affected his mental health. He had experimented with various ketogenic diets including a sardine only, ribeye steak only and a carnivore style of eating as well as periods of extended fasting. He had initially tried prolonged fasting whilst in hospital to help him stay in ketosis, but it was not a sustainable strategy and the food in an acute mental health setting was not ketogenic in nature. He felt his mental health deteriorated because of having to eat higher carbohydrate foods. He now wanted to optimize his KMT and remain in ketosis over a longer period to allow his brain to heal.

## 3 Ketogenic metabolic therapy intervention strategy

Prior to his first acute admission whilst living in a homeless shelter the subject experimented with a ketogenic diet for days at a time. His diet consisted mainly of rib eye steak and minced beef with the occasional egg and avocado interspersed with ‘normal’ high carbohydrate food days. More recently in the rehabilitation situation he began cooking his own ketogenic food. On discharge the subject continued to eat a simplistic ketogenic diet due to his living circumstances. Initially, when staying at a family members house, this consisted mostly of rib-eye steak and mince beef with extra beef tallow. Often, he was only eating one meal a day and supplementing with a lot of MCT oil.

The nutritional therapy practitioner (April 2024) suggested that he needed to eat a minimum of two meals a day to get sufficient protein and energy and gave him specific examples of the amount of meat and fat he needed to consume to stay in stable ketosis. He was advised to eat a 2:1 ratio of fat to protein (there were no carbohydrates as it was a meat and fat only diet). An example of daily meal plan with two meals a day including at least 69 g of protein. Meal suggestions included: a 226 g rib eye steak (43 g protein and 45 g fat) plus an addition 41 g of fat or 200 g of 20% fat beef mince (34 g protein and 40 g fat) plus another 28 g of fat. For fat beef tallow, bone marrow or a similar fat like pork lard was suggested.

It was suggested that he stop supplementing with MCT oil and recommended to drink more water and add a little salt to his food. No other supplementation was used.

The subject tested his ketone/glucose levels twice a day, once in the morning before his first meal of the day and a second time before dinner, 3 h after the last meal. He was recommended to aim for his ketones to be between 2 and 4 mmol/L and no higher than 5 mmol/L. There is limited evidence of a correlation between ketone levels and mental health symptoms suggesting this is an appropriate target range (28).

Compliance with the KMT was monitored using a glucose and ketone blood finger prick testing. Within a couple of days, the subject stabilized his readings with glucose remaining between 4 mmol/L and 6 mmol/L and ketones at between 3.0 mmol/L and 5.0 mmol/L. On a couple of occasions this deviated but he was able to get it quickly back into range. The occasional use of cannabis alongside extended fasting is thought to coincide with the occasional high ketone readings of 8 mmol/L which fits other reports in the literature (29, 30).

The subject’s continued cannabis use resulted in him becoming homeless again, but not due to a psychotic episode, as a result he cut back his cannabis use and focused on remaining mentally stable sustaining a steady state of ketosis. At this point he was living in a hotel room and his diet became more limited, he ate only 20% fat beef mince with beef tallow, cooked in a microwave.

From this point (June 2024) the subject managed by himself without practitioner support. For the following months he stayed consistently in the range of 1 mmol/L to 5 mmol/L still eating a beef mince and tallow carnivore ketogenic diet. There has been a 100% compliance with the diet, he tested glucose and ketones on an almost daily basis throughout the past 9 months from April 2024 to November 2024.

The subject has regularly seen different mental health professionals with little continuity in his care and although interested in his diet they are not able to offer any practical support. His family, who were initially skeptical of the benefits of KMT, now believe it has been more effective than any other therapy.

At around the 7-month mark the subject began to taper off his psychiatric medication. He remained stable throughout this process, the first few days he experienced some insomnia which resolved, and there have been no further adverse side effects.

## 4 Intervention outcomes

The subject was informed in late October 2024 that his mental health team noted his schizophrenia was in remission. He has had no psychotic episodes since he started the ketogenic diet on his discharge from hospital, a period of over nine months. He has come off all his psychiatric medications and remains stable. His community treatment order was discharged in early 2025.

### 4.1 Quantitative analysis

**Figure 1:**
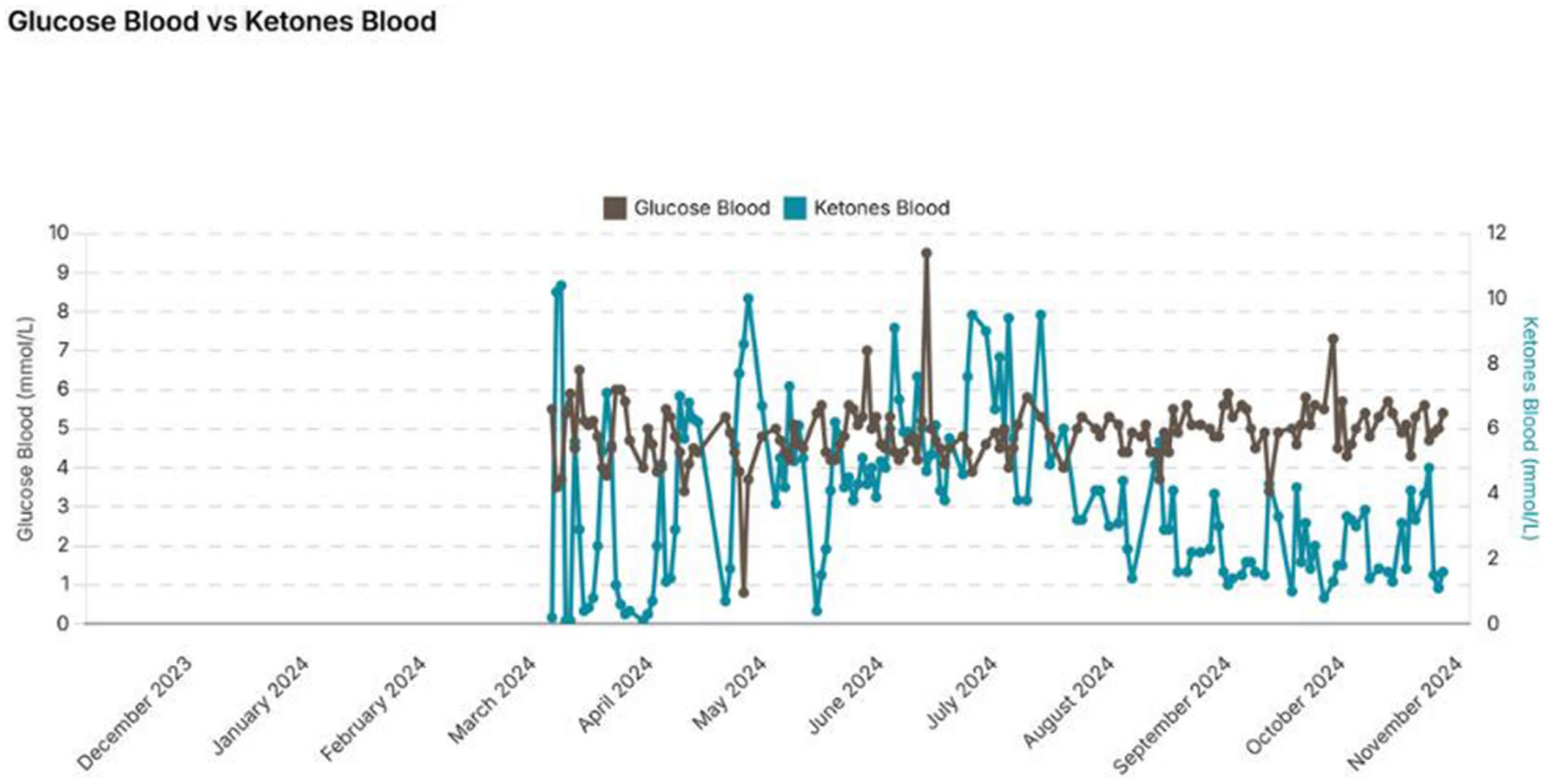
Monitoring of blood glucose and ketones over the 9-month period.

The subject has stayed in steady nutritional ketosis for several months with 100% compliance. This is confirmed by his ketone results and remain between 1.0 and 5 mmol/L but mostly within the 3 to 4 mmol/L level (Figure 1). Over the 9-month period the subject lost a further 5 kg in weight resulting in a body weight of 64 kg and a BMI of 21 which he is happy with.

### 4.2 Qualitative analysis

The subject still suffers from some low mood which he feels this is due to his current socio-economic circumstances. He is awaiting a housing offer and is pleased to have been accepted as a volunteer to help other people with mental health problems.

The subject successfully managed to stick to a carnivore ketogenic diet whilst living in difficult social circumstances, “I would rather eat ground beef and tallow for life than go back into the system.” When in hospital he regularly felt suicidal and wanted to end his own life. He truly believes that if he had not found the ketogenic diet it is likely that he would no longer be alive. He wishes his psychiatric care team had more understanding of the ketogenic diet and felt like he was up against the system trying to give it a go and having no option to use KMT in the acute in-patient setting. He feels that eating a standard high carbohydrate hospital diet worsened his mental health and as a result prolonged his enforced incarceration. He wishes he could have been supported in a facility that enabled him to cook his own food and eat a ketogenic diet with the support of his careers. He thinks that if this had happened, he would have had a faster recovery and been able to integrate back into society and begin volunteering sooner.

## 5 Discussion

The strengths of this case report include the fact that adherence to a ketogenic diet is confirmed from daily ketone and glucose measurements and despite receiving only limited professional support, the subject was able to maintain the diet over several months. This success was likely facilitated by his strong motivation to avoid rehospitalization and his substantial knowledge of ketogenic dietary practices.

Limitations include the lack of quantitative clinical data such as scores from validated tools (e.g., GAD-7, PTSD-related scales, or other clinical psychiatric evaluations). These are essential for a more objective assessment of mental health outcomes and would significantly strengthen the case report’s rigor.

There is a lack of research on carnivore ketogenic diets in general and whether they result in any long-term nutrient deficiencies or adverse effects on the gut microbiome. To date one long term study has sought to compare the microbiome of an individual consuming a strict carnivore diet for 4 years with controls consuming varying quantities of meat, including no meat, findings were that there was no difference in the gut microbiome (31). In addition, studies investigating the nutrient content and deficiency of carnivore diets found mixed results. (37) found that four versions of the carnivore diet achieved most nutrient reference values with several falling short including thiamine, magnesium, calcium, and Vitamin C, and iron, folate, iodine, and potassium in some cases. O’Hearn (32) argues all essential nutrients can be found in animal foods and that requirements for nutrient intake may reduce on a carnivore diet; with studies on individuals consuming a carnivore diet not demonstrating deficiencies, this theory remains unproven. An example of this is vitamin C which competes for uptake with glucose (33) and that meat has been used traditionally to prevent scurvy despite being a poor source of vitamin C (Geodeke et al., 2024).

Further limitations include the fact that no precise control of macronutrient intake was possible, and it is possible that the subject deviated from the suggested protein and fat recommendations although ketone levels suggest this was not the case. Despite high fat diets traditionally being associated with cardiovascular disease a 2024 review considering diet alongside conventional and emerging physiological risk factors and found there was no evidence to support a link between total fat intake and risk of coronary heart disease or CVD mortality (34). Raised LDL levels can occur in a subset of individuals on a ketogenic diet but alongside an improvement in most lipid marks this does not necessarily place a person at an elevated risk for CVD (35).

It is possible that removal of gluten from the diet could have been a key factor in the subject achieving remission (36). A carnivore ketogenic diet is a more restrictive diet and can be more difficult to adhere to than other forms of ketogenic diets suggesting that its use as a therapeutic tool may be limited. There are many factors potentially affecting long-term adherence to a ketogenic diet including socioeconomic and cultural environments as well as potential addiction to ultra-processed foods and lack of access to real food. It is also unknown whether a ketogenic diet is required for life or if the brain can heal over sufficiently over time therefore enabling a return to a healthy non-ketogenic diet. As this is the first case report of an individual using a carnivore ketogenic diet for schizophrenia there is insufficient evidence available to suggest that this will be a helpful strategy for other people with a similar diagnosis.

From a patient perspective information and support for this choice of therapy needs to be made available. As this case report has demonstrated it can be life changing and lifesaving. Registered nutritional therapy practitioners can play a role working alongside mental health practitioners to support people with personalized dietary changes. Peer support or health coaching might offer cost effective solutions to enable health services to embrace small pilots of ketogenic therapy alongside mainstream services.

## 6 Summary

This case report demonstrates that even in difficult social circumstances and with limited professional support it is potentially possible to implement a ketogenic metabolic therapy and put serious mental illness into remission. It suggests that a carnivore ketogenic diet may be one option to help individuals improve brain function, resolve mental health symptoms and restore daily life functioning. It raises the potential for nutritional therapy practitioners to work alongside mental health professionals to help subjects implement and sustain ketogenic diets.

## Data Availability

The original contributions presented in the study are included in the article/supplementary material, further inquiries can be directed to the corresponding author.

## References

[1] Di Luzio M Pontillo M Villa M Attardi AG Bellantoni D Di Vincenzo C. Clinical features and comorbidity in very early-onset schizophrenia: a systematic review. Front Psych. (2023) 14:1270799. doi: 10.3389/fpsyt.2023.1270799, PMID: 38152354 PMC10752227

[2] Navti B Nikolic N. Current treatments in schizophrenia. Prescriber. (2024) 35:9–16. doi: 10.1002/psb.2147

[3] Nevarez-Flores AG Sanderson K Breslin M Carr VJ Morgan VA Nei l AL. Systematic review of global functioning and quality of life in people with psychotic disorders. Epidemiol Psychiatr Sci. (2019) 28:31–44. doi: 10.1017/S2045796018000549 30270819 PMC7179814

[4] Tandon R Nasrallah H Akbarian S Carpenter WT Jr DeLisi LE Gaebel W. The schizophrenia syndrome, circa 2024: what we know and how that informs its nature. Schizophr Res. (2023) 264:1–28. doi: 10.1016/j.schres.2023.11.015 38086109

[5] Sarnyai Z Ben-Shachar D. Schizophrenia, a disease of impaired dynamic metabolic flexibility: a new mechanistic framework. Psychiatry Res. (2024) 342:116220. doi: 10.1016/j.psychres.2024.116220, PMID: 39369460

[6] Annamalai A Kosir U Tek C. Prevalence of obesity and diabetes in patients with schizophrenia. World J Diabetes. (2017) 8. doi: 10.4239/wjd.v8.i8.390, PMID: 28861176 PMC5561038

[7] Mikkelsen TJ Agerskov H Jensen DM Stenager E Rothmann MJ. Living with schizophrenia and type 2 diabetes and the implication for diabetes self-care: a qualitative study. J Clin Nurs. (2024) 33:1862–1874. doi: 10.1111/jocn.17001, PMID: 38356190

[8] Guest PC. Insulin Resistance in Schizophrenia. Adv Exp Med Biol. (2019) 1134. doi: 10.1007/978-3-030-12668-1_1, PMID: 30919329

[9] Anderson J Ozan E Chouinnard V Crant G MacDonald A Thakkar L. The ketogenic diet as a transdiagnostic treatment for neuropsychiatric disorders: mechanisms and clinical outcomes. Curr Treat Options Psychiatry. (2024) 12:1. doi: 10.1007/s40501-024-00339-4

[10] Ferraris C Guglielmetti M Neri LCL Allehdan S Albasara JMM Fareed Alawadhi HH. A review of ketogenic dietary therapies for epilepsy and neurological diseases: a proposal to implement an adapted model to include healthy Mediterranean products. Food Secur. (2023) 12:12091743. doi: 10.3390/foods12091743 PMC10178865 37174282

[11] Laurent N Bellamy EL Hristova D Houston A. Ketogenic diets in clinical psychology: examining the evidence and implications for practice. Front Psychol. (2024) 15. doi: 10.3389/fpsyg.2024.1468894, PMID: 39391844 PMC11464436

[12] Vidali S Aminzadeh S Lambert B Rutherford T Sperl W Kofler B. Mitochondria: the ketogenic diet--a metabolism-based therapy. Int J Biochem Cell Biol. (2015) 63:55–9. doi: 10.1016/j.biocel.2015.01.022, PMID: 25666556

[13] Saris CGJ Timmers S. Ketogenic diets and ketone supplementation: a strategy for therapeutic intervention. Front Nutr. (2022) 9. doi: 10.3389/fnut.2022.947567 PMC9705794 36458166

[14] Paol i A Bianco A Moro T Mota JF Coelho-Ravagnani CF. The effects of ketogenic diet on insulin sensitivity and weight loss, which came first: the chicken or the egg? Nutrients. (2023) 15. doi: 10.3390/nu15143120 PMC10385501 37513538

[15] Danan A Westman EC Saslow LR Ede G. The ketogenic diet for refractory mental illness: a retrospective analysis of 31 inpatients. Fron t Psychiatry. (2022) 13. doi: 10.3389/fpsyt.2022.951376, PMID: 35873236 PMC9299263

[16] Palmer CM Gilbert-Jaramillo J Westman EC. The ketogenic diet and remission of psychotic symptoms in schizophrenia: two case studies. Schizophr Res. (2019) 208:439–40. doi: 10.1016/j.schres.2019.03.019, PMID: 30962118

[17] Sethi S Wakeham D Ketter T Hooshmand F Bjornstad J Richards B. Ketogenic diet intervention on metabolic and psychiatric health in bipolar and schizophrenia: a pilot trial. Psychiatry Res. (2024) 335:115866. doi: 10.1016/j.psychres.2024.115866, PMID: 38547601

[18] Laurent N Bellamy EL Tague KA Hristova D Houston A. Ketogenic metabolic therapy for schizoaffective disorder: a retrospective case series of psychotic symptom remission and mood recovery. Front Nutr. (2025) 12:1506304. doi: 10.3389/fnut.2025.1506304, PMID: 39990610 PMC11844221

[19] Rangaraju V Lewis TL Jr Hirabayashi Y Bergami M Motori E Cartoni R. Pleiotropic mitochondria: the influence of mitochondria on neuronal development and disease. J Neurosci. (2019) 39:8200–8. doi: 10.1523/JNEUROSCI.1157-19.2019, PMID: 31619488 PMC6794931

[20] Clay HB Sillivan S Konradi C. Mitochondrial dysfunction and pathology in bipolar disorder and schizophrenia. Int J Dev Neurosci. (2011) 29:311–24. doi: 10.1016/j.ijdevneu.2010.08.007 20833242 PMC3010320

[21] Gubert C Stertz L Pfaffenseller B Panizzutti BS Rezin GT Massuda R. Mitochondrial activity and oxidative stress markers in peripheral blood mononuclear cells of patients with bipolar disorder, schizophrenia, and healthy subjects. J Psychiatr Res. (2013) 47:1396–402. doi: 10.1016/j.jpsychires.2013.06.018, PMID: 23870796

[22] Więdłocha M Zborowska N Marcinowicz P Dębowska W Dębowska M Zalewska A. Oxidative stress biomarkers among schizophrenia inpatients. Brain Sci. (2023) 13:490. doi: 10.3390/brainsci13030490, PMID: 36979300 PMC10046541

[23] Dobersek U Wy G Adkins J Altmeyer S Krout K Lavie CJ. Meat and mental health: a systematic review of meat abstention and depression, anxiety, and related phenomena. Crit Rev Food Sci Nutr. (2021) 61. doi: 10.1080/10408398.2020.1741505, PMID: 32308009

[24] Dobersek U Bender M Etienne A Fernandez Gil GE Hostetter C. Meat consumption & positive mental health: a scoping review. Prev Med Rep. (2023) 37:102556. doi: 10.1016/j.pmedr.2023.102556 38186660 PMC10770626

[25] Jacka FN Pasco JA Williams LJ Mann N Hodge A Brazionis L. Red meat consumption and mood and anxiety disorders. Psychother Psychosom. (2012) 81:196–8. doi: 10.1159/000334910, PMID: 22433903

[26] Lennerz BS Mey JT Henn OH Ludwig DS. Behavioral characteristics and self-reported health status among 2029 adults consuming a "carnivore diet". Curr Dev Nutr. (2021) 5:nzab133. doi: 10.1093/cdn/nzab133, PMID: 34934897 PMC8684475

[27] Ramesh R Kanagasingam A Sabrina S Anushanth U. Starvation ketoacidosis in a young healthy female after prolonged religious fasting. Cureus. (2023) 15:e39962. doi: 10.7759/cureus.39962, PMID: 37416003 PMC10320649

[28] Needham N Campbell IH Grossi H Kamenska I Rigby BP Simpson SA. Pilot study of a ketogenic diet in bipolar disorder. BJ Psych Open. (2023) 9:e176. doi: 10.1192/bjo.2023.568, PMID: 37814952 PMC10594182

[29] Pinto JS Martel F. Effects of Cannabidiol on appetite and body weight: a systematic review. Clin Drug Investig. (2022) 42:909–19. doi: 10.1007/s40261-022-01205-y, PMID: 36180814 PMC9525229

[30] Spanagel R Bilbao A. Approved cannabinoids for medical purposes - comparative systematic review and meta-analysis for sleep and appetite. Neuropharmacology. (2021) 196:108680. doi: 10.1016/j.neuropharm.2021.108680, PMID: 34181977

[31] Karačić A Dikarlo D Dorst I Renko I Liberati Pršo A. The gut microbiome without any plant food? A case study on the gut microbiome of a healthy carnivore. Microbiota Host. (2024) 2:3120. doi: 10.1530/MAH-24-0006

[32] O'Hearn A. Can a carnivore diet provide all essential nutrients? Curr Opin Endocrinol Diabetes Obes. (2020) 27:312–6. doi: 10.1097/MED.0000000000000576, PMID: 32833688

[33] Chen L Jia RH Qiu CJ Ding G. Hyperglycemia inhibits the uptake of dehydroascorbate in tubular epithelial cell. Am J Nephrol. (2005) 25:459–65. doi: 10.1159/000087853, PMID: 16118484

[34] Ma Y Zheng Z Zhuang L Wang H Li A Chen L. Dietary macronutrient intake and cardiovascular disease risk and mortality: a systematic review and dose-response Meta-analysis of prospective cohort studies. Nutrients. (2024) 16:152. doi: 10.3390/nu16010152, PMID: 38201983 PMC10780780

[35] Diamond DM Mason P Bikman BT. Opinion: are mental health benefits of the ketogenic diet accompanied by an increased risk of cardiovascular disease? Front Nutr. (2024) 11:1394610. doi: 10.3389/fnut.2024.1394610, PMID: 38751739 PMC11095042

[36] Kelly DL Demyanovich HK Rodriguez KM Ciháková D Talor MV McMahon RP. Randomized controlled trial of a gluten-free diet in patients with schizophrenia positive for antigliadin antibodies (AGA IgG): a pilot feasibility study. J Psychiatry Neurosci. (2019) 44:269–76. doi: 10.1503/jpn.180174 30938127 PMC6606425

[37] Goedeke S Murphy T Rush A Zinn C. Assessing the nutrient composition of a carnivore diet: A case study model. Nutr. (2024) 17:140. doi: 10.3390/nu17010140 PMC11722875 39796574

